# PARP inhibitors in ovarian cancer

**DOI:** 10.1038/bjc.2015.392

**Published:** 2015-12-15

**Authors:** David Cibula, Judith Balmaña

**Affiliations:** 1 Gynecologic Oncology Center, Department of Obstetrics and Gynecology, General University Hospital in Prague, First Faculty of Medicine, Charles University, Prague, Czech Republic; 2 Medical Oncology Department, Vall d‘Hebron University Hospital, Vall d‘Hebron Institute of Oncology (VHIO), Barcelona, Spain

The fact that most women diagnosed with ovarian cancer initially present in the advanced stages of the disease constitutes one of the greatest barriers to effective treatment. The failure of existing treatments is largely reflected by the fact that ovarian cancer remains the most common cause of gynaecological cancer death, with rates of survival in the UK among the worst in Western Europe (Ovarian Cancer Action, 2014). Cytoreductive surgery followed by platinum-based chemotherapy, while it represents the standard of care for advanced ovarian cancer, does not appear to adequately maintain disease control at 5 years, or prevent recurrence in most patients (Jayson *et al*, 2014). With these failings in mind, a tailored approach to treatment – exemplified by targeted therapies and selection of the patient population based on biomarker or genetic screening – seems to currently be a promising emerging strategy to improve survival in women with ovarian cancer.

In this supplement, articles by Ledermann *et al* and Drew comprehensively review molecular and clinical evidence underlying the evolution of poly (ADP-ribose) polymerase (PARP) inhibitors in a journey spanning 50 years, which culminates in the recent licensing approval of the first oral PARP inhibitor, olaparib (Lynparza) for *BRCA*-mutated high-grade serous ovarian cancer. In addition, George discusses current UK procedures for *BRCA* testing of at-risk women and considers new genetic testing models in development.

The emergence of the DNA repair pathway as a rational target in various cancers led to the development of the PARP inhibitors. A novel class of agent, PARP inhibitors exploit the mechanism of ‘synthetic lethality’ (loss of function in two genes causes cell death as opposed to a non-lethal effect of functional loss of one of the genes) to target tumours with defective DNA repair mechanisms, such as aberrant homologous recombination repair due to loss of *BRCA1* and *2* gene function through the presence of mutations. As reviewed by Drew in this supplement, *in vitro* and *in vivo* studies have shown that cells deficient in *BRCA1* and *2* are more sensitive to PARP inhibition than proficient cells. Furthermore, 10–15% of women with ovarian cancer are reported to carry an inherited germline *BRCA* mutation (g*BRCA*m). Indeed, when describing the history of PARP inhibitors and their interaction with *BRCA1* and *2* genes, Drew notes the increasing recognition of *BRCA*m status as not only a marker of familial risk, but also a major determinant of treatment. An important point to consider and which has marked implications for treatment outcome is that the number of *BRCA*m carriers among women with ovarian cancer may be significantly underestimated, particularly in those with high-grade serous ovarian cancer and no family history of cancer. In this regard, it has been shown that approximately 50% of patients with ovarian cancer, and who are g*BRCA*m carriers, had no family history of ovarian or breast cancer and might not be considered for germline genetic testing under current clinical guidelines (Alsop *et al*, 2012). From this viewpoint, the interaction between PARP inhibitors and *BRCA* mutations holds great promise for a tailored treatment strategy in the ovarian cancer setting. It should also be noted that in addition to mutations to the *BRCA1/2* genes, which account for approximately two-thirds of germline mutations in ovarian cancer, germline mutations in a range of genes associated with DNA repair mechanisms are associated with an inherited predisposition to the disease (Senter *et al*, 2008; Bonadona *et al*, 2011; Loveday *et al*, 2011; Pelttari *et al*, 2011; Rafnar *et al*, 2011; Loveday *et al*, 2012; Turnbull *et al*, 2014; Xiao *et al*, 2014). Furthermore, as discussed by George, somatic mutations in a range of different genes have been associated with each subtype of epithelial ovarian cancer. Interest in this range of genes may increase if they are found to influence response to targeted treatments and chemotherapy.

The theoretical advantages of personalised treatment cannot be disputed, although the practical aspects of patient selection and determining delivery and optimal timing of *BRCA* testing present challenges to healthcare organisations worldwide. Taking the USA as an example, family history of ovarian or breast cancer is maintained as the basis for the most recent guidance from the United States Preventive Services Task Force on risk assessment and *BRCA* testing of women, but no provision is made for testing of women with ovarian or breast cancer at diagnosis (Moyer, 2014). Meanwhile, the National Comprehensive Cancer Network (NCCN) guidelines were updated in 2014 (National Comprehensive Cancer Network, 2014) to recommend genetic risk evaluation (*BRCA*m testing) in patients with a suspicious/palpable pelvic mass and the Ovarian Cancer Action BRCA1/2 Gene Policy Report published early 2014 served as a call to action for the UK government to recognise that all women with the non-mucinous subtype of epithelial ovarian cancer should be tested for *BRCA* mutations at the point of diagnosis, given that around half of these women do not have a family history of the disease (Ovarian Cancer Action, 2014). With this in mind, there is the need to establish correct processes in the clinical management pathway to accommodate genetic testing of women with ovarian cancer, in order not to overlook *BRCA*m carriers who may have potentially benefited from targeted treatment.

In their review, Ledermann *et al* provide an overview of clinical trial data for olaparib in ovarian cancer and conclude that patients likely to derive the greatest benefit from PARP inhibition are those with platinum-sensitive relapsed ovarian cancer and a *BRCA*m. Key supporting data comprised results from a pivotal Phase II maintenance trial (Study 19) of more than 200 patients with ovarian cancer, and documented response to platinum-based chemotherapy, which revealed a significant improvement in the primary endpoint of progression-free survival (PFS) with olaparib *vs* placebo (Ledermann *et al*, 2012). Additional pre-planned *post-hoc* analyses of the Phase II data by *BRCA*m status showed a significant clinical benefit of olaparib in women with a *BRCA*m in terms of prolonged PFS and delay in time to first or second subsequent therapy or death (Ledermann *et al*, 2014). On the basis of the Phase II study findings and other clinical trial data, the capsule formulation of olaparib has recently been approved in Europe for platinum-sensitive relapsed *BRCA*m high-grade serous ovarian cancer in response to platinum-based chemotherapy, and in the USA as monotherapy for patients with advanced relapsed high-grade serous ovarian cancer with a deleterious/suspected deleterious g*BRCA*m who have received three or more prior lines of chemotherapy. Results are also awaited from two Phase III studies, SOLO1 (NCT01844986) and SOLO2 (NCT01874353), which are underway for evaluation of a new tablet formulation of olaparib for the maintenance treatment of ovarian cancer in patients with a *BRCA*m. The second of these trials, SOLO2, will evaluate whether a broader population of patients with *BRCA*m ovarian cancer (i.e., patients with high-grade endometrioid ovarian cancer, as well as those with high-grade serous disease) will benefit from treatment with olaparib.

Thus, the clinical validity of PARP inhibitor-mediated treatment of *BRCA*m ovarian cancer has been formally recognised by licensing authorities. However, the question remains whether the appropriate management steps and procedures are in place to enable extrapolation of these findings to real-world practice. *BRCA* testing in women with ovarian cancer and challenges surrounding its implementation are discussed in detail by George, who highlighted several problematic issues in UK genetic testing procedures: under-referral for testing, lack of a unified *BRCA* testing process and no appropriate guideline provision for somatic *BRCA*m testing. Once these limitations are recognised, initiatives that successfully help to accommodate *BRCA*m testing will positively impact the proportion of women able to gain benefit from targeted treatment.

In summary, in the evolving treatment landscape for ovarian cancer, the PARP inhibitor olaparib is emerging as a rational therapy that offers a realistic opportunity to achieve effective disease control through targeted and personalised treatment.
